# Next Generation Risk Assessment of Acute Neurotoxicity from Organophosphate Exposures Using the In Vitro–In Silico Derived Dietary Comparator Ratio

**DOI:** 10.1021/acs.est.5c00220

**Published:** 2025-03-19

**Authors:** Jiaqi Chen, Thijs M. J. A. Moerenhout, Nynke I. Kramer, Ivonne M. C. M. Rietjens

**Affiliations:** Division of Toxicology, Wageningen University and Research, Stippeneng 4, 6708 WE Wageningen, The Netherlands

**Keywords:** organophosphate pesticides, acetylcholinesterase inhibition, Dietary Comparator Ratio, Next Generation Risk Assessment, 3Rs-compliant approach

## Abstract

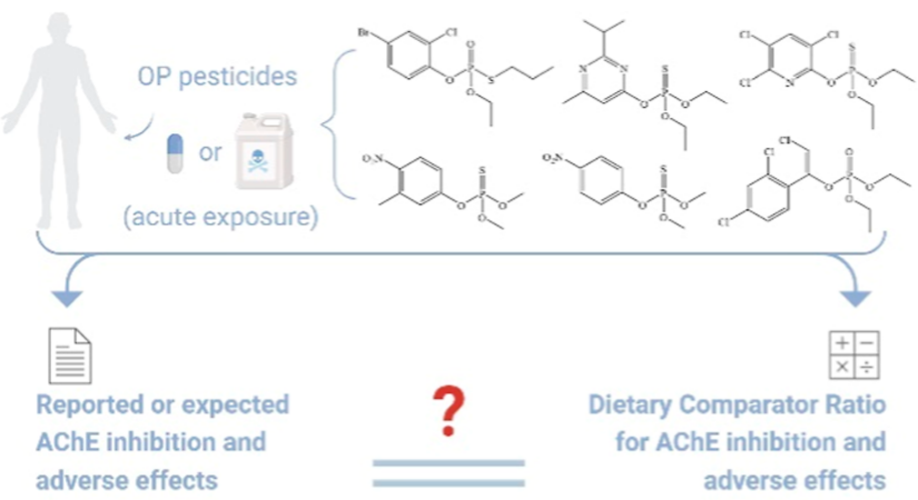

Organophosphate (OP) pesticides are common environmental contaminants, of which the resulting acetylcholinesterase (AChE) inhibition and concomitant neurotoxic effects following exposure remain a global concern. To evaluate the safety upon acute exposure to OP pesticides, the Dietary Comparator Ratio (DCR) approach was used for the first time for this class of chemicals. Six OPs including chlorpyrifos, diazinon, fenitrothion, methyl parathion, profenofos, and chlorfenvinphos were selected as model compounds. Seventy-four reports of human exposures were collected, and a DCR value at each defined exposure level was calculated with in vitro determined AChE inhibition potency and in silico simulated internal exposures. Results indicate that the DCR outcomes are comparable to the actual knowledge on the presence or absence of in vivo AChE inhibition and adverse effects for the respective exposure scenarios. Of all collected scenarios, only four false positives but no false negatives were obtained. No safety concern on acute neurotoxicity appears to be raised for the evaluated environmental exposure scenarios to OPs. To conclude, the described DCR approach provides an adequate evaluation of the OP-induced adverse outcomes for humans, shedding light on its utility for 3Rs-compliant safety assessment of chemicals with different toxicity mechanisms especially for which in vitro bioassays are available.

## Abstract 2

With an appropriate comparator, the Dietary Comparator Ratio approach based on data from in vitro bioassays and in silico predictions is promising for human safety assessment of environmental chemical exposures.

## 1 Introduction

**Figure 1:**
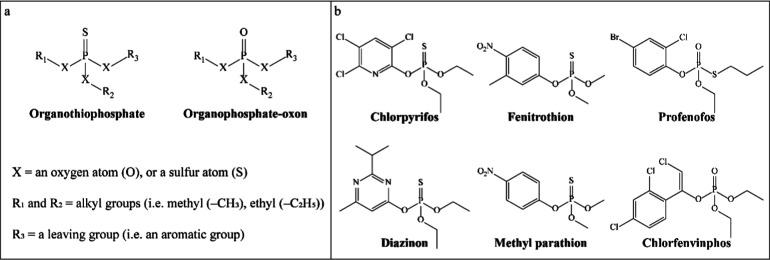
Chemical structures of (a) organothiophosphates and organophosphate oxons, and (b) the OP pesticides selected as model compounds in this work.

Organophosphate (OP) pesticides have been used for pest control since the 1940s.^1,2^ In spite of the ban on several OP pesticides in various countries and regions, extensive application of OP pesticides results in residues being continuously found in food products and the environment.^3−5^ In addition, OP pesticides and related metabolites have also been detected in human urine and breast milk as reported in biomonitoring investigations.^6−8^ Dietary ingestion of OP pesticides via food and drinking water along with accidental and intentional OP exposures among the general population remains a global public health issue,^9,10^ demanding a risk evaluation for such exposure scenarios. OP pesticides can be broadly categorized into two groups, organothiophosphates and organophosphate oxons, containing a phosphoryl–sulfur double bond (P=S) or a phosphoryl–oxygen double bond (P=O), respectively (Figure 1a). With an oxidative desulfuration of the thiophosphate group mediated primarily by hepatic cytochrome P450 (CYP450), an organothiophosphate can be bioactivated to the corresponding organophosphate-oxon analogue.^11^ Different from organophosphate oxons, organothiophosphates themselves exert limited inhibition toward acetylcholinesterase (AChE) in humans.^11,12^ Therefore, the OP-induced acute neurotoxicity is mainly attributed to the irreversible binding between neuronal AChE and the organophosphate oxon, hindering the breakdown of acetylcholine at the synaptic cleft and causing cholinergic toxidrome in mammals.^13^

For chemical risk assessment, large strides have been made in the past decades in developing in vitro toxicity testing to accelerate the transformation from the conventional means that depend heavily on animals to Next Generation Risk Assessment minimizing reliance on in vivo testing.^14,15^ While in vitro toxicity assays, such as the in vitro AChE inhibition assay, are powerful tools to develop mechanistic insights into the chemical potency,^16^ implementation of these assays in risk assessment still remains elusive due to a gap between in vitro toxicity data sets and in vivo adverse outcomes. To bridge the gap, the Dietary Comparator Ratio (DCR) approach has been proposed for chemical risk assessment, considering mode of action, toxicity potency, and exposure context.^17^ This approach is based on the use of a dietary compound with a history of safe consumption as a point of reference (the comparator). However, in essence, every compound can serve as a comparator compound as long as a safe in vivo exposure level can be defined.

The DCR approach works for chemicals with the same mode of action and evaluates the Exposure Activity Ratio (EAR) of an exposure scenario for a test substance (EAR_test_) along with the EAR of a safe human exposure to a comparator compound (EAR_comparator_). Calculated as the ratio of the EAR_test_ against the EAR_comparator_, the resultant DCR is then used to evaluate the safety of the exposure scenario for the test compound, where a DCR value below 1 indicates a safe exposure scenario.^18−20^ The EARs can be defined with in vitro derived toxicity information and in silico simulated internal exposure data,^17^ hence, no animal testing is required when employing the DCR approach for chemical risk evaluation. This approach has been proved as adequate for safety assessment of chemically induced antiandrogenicity and estrogenicity,^18−20^ and it is of interest to explore its utility for chemicals with other modes of action.

The aim of the current study is to investigate the feasibility of applying the DCR approach for the safety evaluation of acute human exposure to OP pesticides. Six OPs including four organothiophosphates (chlorpyrifos, diazinon, fenitrothion, and methyl parathion) and two organophosphate oxons (profenofos and chlorfenvinphos) (Figure 1b) were selected as model compounds. For the model OPs, relevant data on available human exposures and the resulting effects were collected from the literature. Because of the high sensitivity and accessibility, erythrocyte AChE inhibition was used as a surrogate end point to the inhibition of neuronal AChE,^21^ and an in vitro assay with human blood samples was performed to assess the inhibitory potential of the selected compounds and/or the active oxon metabolites. Together with the scenario-specific internal oxon levels predicted by a recently developed generic human physiologically based kinetic (PBK) model,^22^ the EAR_test_ and EAR_comparator_ were defined and used for DCR calculations at the defined exposure levels. The obtained DCR outcomes were compared to the actual knowledge of health outcomes (AChE inhibition and adverse health effects) reported for the respective exposure scenarios, enabling the evaluation of the DCR approach and providing insights into its utility for predicting safe thresholds of human exposure to OP pesticides.

## 2 Materials and Methods

### 2.1 Chemical and Biological Materials

Methyl paraoxon, profenofos, chlorfenvinphos, acetylthiocholine iodide (ATC), 5,5′-dithiobis(2-nitrobenzoic acid) (DTNB), ethopropazine, NaH_2_PO_4_, Na_2_HPO_4_, and Triton-X 100 were purchased from Sigma-Aldrich (Amsterdam, The Netherlands). Chlorpyrifos oxon and diazoxon were ordered from TRC-Canada (Toronto, Ontario, Canada). Ethanol (UPLC/MS grade) was ordered from Biosolve (Valkenswaard, The Netherlands). Ultrapure water used for experiments was prepared with a Sartorius Arium Pro ultrapure water system (Göttingen, Germany). Human whole peripheral blood with K_2_EDTA as an anticoagulant was used for the in vitro AChE inhibition assay. K_2_EDTA acts as an inhibitor to the paraoxonase 1 (PON1) enzyme,^23^ preventing the underestimation of the inhibition potential of organophosphate oxons due to the nontarget clearance by PON1 present in the blood samples.

### 2.2 Preparation and Application of the DCR Approach

The workflow of the DCR approach employed in the present study for risk assessment of acute exposure to OP pesticides was modified based on the previous applications for antiandrogens and estrogens^18−20^ and is described step by step in the following sections.

#### 2.2.1 Step 1: Selection of Model Compounds

OP pesticides, including both organothiophosphates and organophosphate oxons, were selected as model compounds, based on the availability of literature data on human exposure and of a generic human PBK model for predicting internal concentrations following exposure,^22^ as these are necessary for the application of the DCR approach.^18−20^

#### 2.2.2 Step 2: Collection of In Vitro Concentration–Response Data Using the AChE Inhibition Assay

Inhibition of erythrocyte AChE by the organophosphate oxon or the corresponding oxon metabolite of an organothiophosphate was measured in vitro using the method previously described.^12^ The ethanolic stock solution of the test oxon was first diluted 100 times with sodium phosphate buffer (100 mM, pH 7.4), and 10 μL of the diluted solution was added to 90 μL of human whole blood sample in a 48-wells flat-bottom transparent plate (Greiner Bio-One, The Netherlands). After a 30 min incubation at room temperature, the blood samples were then diluted 20 times with ultrapure water containing 0.03% Triton-X 100 (v/v), and 20 μL of the Triton-diluted blood was incubated for 20 min following the addition of 460 μL of DTNB and 20 μL of ethopropazine (an inhibitor to plasma butyrylcholinesterase (BuChE)). After that, 100 μL of ATC was added (final concentrations of ethopropazine, DTNB, and ATC were 20 μM, 1 mM, and 1 mM, respectively), and the absorbance at 436 nm was measured continuously to detect the remaining AChE activity. Controls were carried out by replacing the test compound with sodium phosphate buffer to correct for any background absorbance. Assays were conducted in triplicate, and the obtained data were analyzed using a nonlinear regression of the log(inhibitor) vs response–variable slope (four parameters) model in GraphPad Prism (version 5.04, San Diego, CA, USA) to define the concentration resulting in 50% erythrocyte AChE inhibition (IC_50_).

Additionally, for the comparator compound, the lower 95% confidence limit of the benchmark concentration associated with 5% erythrocyte AChE inhibition (BMCL_05_) was determined by performing benchmark concentration (BMC) analysis on the obtained in vitro AChE inhibition data. The benchmark response (BMR) of 5% for erythrocyte AChE inhibition is a conservative end point for deriving an effect level, since regulatory bodies usually use a higher BMR (i.e., 10% or 20%) for risk assessment of acute OP pesticide exposure.^24,25^ The European Food Safety Authority (EFSA) Web tool (https://efsa.openanalytics.eu/) integrated with the R package PROAST (version 70.0) was employed for the BMC analysis (see Supporting Information).

#### 2.2.3 Step 3: Collection of Available Exposure Scenarios and Relevant Adverse Health Effects

Acute exposure scenarios where human individuals were orally exposed to one of the model OPs were gathered from the literature. The collected scenarios could be broadly classified into two categories: human volunteer studies at relatively low dose levels, where adverse effects might not be observed, and intentional and/or accidental exposures, for which usually a relatively high OP pesticide dose was ingested and the resulting AChE inhibition and/or adverse health effects were reported. Data on the first oral administration in repeated dose studies were also collected and considered to represent an acute exposure scenario. Based on the information on erythrocyte AChE inhibition and the resultant adverse health effects at respective exposure scenarios, it could be concluded whether the respective dose levels induced a positive, negative, or unknown (unreported) adverse effect in humans. This enabled the evaluation of the obtained DCR outcomes in step 7 (see Section 2.2.7).

#### 2.2.4 Step 4: Prediction of Internal Concentrations for Collected Exposure Scenarios

A newly developed generic human PBK model for OP pesticides was used to predict the maximum blood oxon concentration (C*B*_max_oxon) for the collected exposure scenarios,^22^ considering that this value is relevant to the erythrocyte AChE inhibition induced by acute exposure to an OP pesticide.^26,27^ No corrections for differences in in vivo and in vitro protein binding are necessary, since the in vitro AChE inhibition assay was performed with human blood samples (see Section 2.2.2).

When predicting C*B*_max_oxon with the generic PBK model,^22^ dose levels and oral absorption rate constants were needed as model inputs. Generally, in volunteer studies, the tested dose levels (in milligrams per kilogram of BW) are explicitly reported, while this information is unclear for most accidental and intentional exposures. For such scenarios, the exposure level was estimated with the ingested amount (in mg) assuming a body weight of 70 kg regardless of age, sex, and ethnicity, unless a specific body weight was reported. With regard to the oral absorption rate constant (in h^–1^), OP-specific values calculated based on quantitative structure–activity relationships (QSARs) are available^22,28^ and are summarized in Table S1 (see Supporting Information). For C*B*_max_oxon prediction, both the established rat and human generic PBK models have been reported to adequately predict internal exposure of OP pesticides, while it is important to note that the rat model has been reported to overpredict the blood oxon concentration for dimethyl-organothiophosphates including fenitrothion and methyl parathion by about 1 order of magnitude.^22^ Since no relevant data on the blood oxon profile following exposure to fenitrothion or methyl parathion are available to evaluate the human model, the assumption of the same overprediction magnitude (10-fold) was made in this work. Therefore, the C*B*_max_oxon predictions from the generic human PBK model for fenitrothion and methyl parathion were divided by 10 to correct for the probable overestimation.

#### 2.2.5 Step 5: Selection of a Comparator Compound

Single oral chlorpyrifos exposure scenarios that did not result in notable adverse health effects in humans are available and well-described,^21^ making this organothiophosphate an adequate comparator. The generic human PBK model was used to predict the C*B*_max_oxon under a safe chlorpyrifos dose level, and the in vitro derived BMCL_05_ was compared with this predicted value to determine if the BMCL_05_ could indeed represent a safe scenario to define the EAR_comparator_.

#### 2.2.6 Step 6: DCR Calculation for Collected Exposure Scenarios

The DCR value for an exposure scenario was calculated as the ratio of EAR_test_ and EAR_comparator_ (eq 1). The EAR_test_ is scenario-specific and was defined with the IC_50_ of the tested organophosphate oxon for in vitro erythrocyte AChE inhibition and the PBK model-simulated C*B*_max_oxon at the defined exposure level (eq 2). Chlorpyrifos was selected as the comparator compound, and the EAR_comparator_ was calculated using the BMCL_05_ and IC_50_ derived from the in vitro AChE inhibition assay for chlorpyrifos oxon (eq 3).
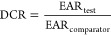
1
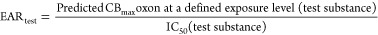
2
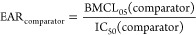
3

#### 2.2.7 Step 7: Evaluation and Application of the DCR Approach

The DCR-based predictions were evaluated by comparing the obtained DCR results with actual knowledge of the adverse health effects reported for the respective exposure scenarios. Adverse effects cannot be ruled out for human exposure scenarios with a DCR result greater than 1, while exposure scenarios with a DCR result less than or equal to 1 are considered to raise no safety concern.^18−20^ Once validated as an adequate predicting tool, the DCR approach was applied for evaluating the safety of exposure scenarios, for which no information on the resulting toxic outcomes was reported at the defined dose levels for humans.

## 3 Results

### 3.1 Step 1: Selection of Model Compounds

Six OP pesticides were selected as model compounds, including two diethyl-organothiophosphates (chlorpyrifos and diazinon), two dimethyl-organothiophosphates (fenitrothion and methyl parathion), and two organophosphate oxons (profenofos and chlorfenvinphos) (Figure 1b).

### 3.2 Step 2: Collection of In Vitro Concentration–Response Data Using the AChE Inhibition Assay

**Figure 2:**
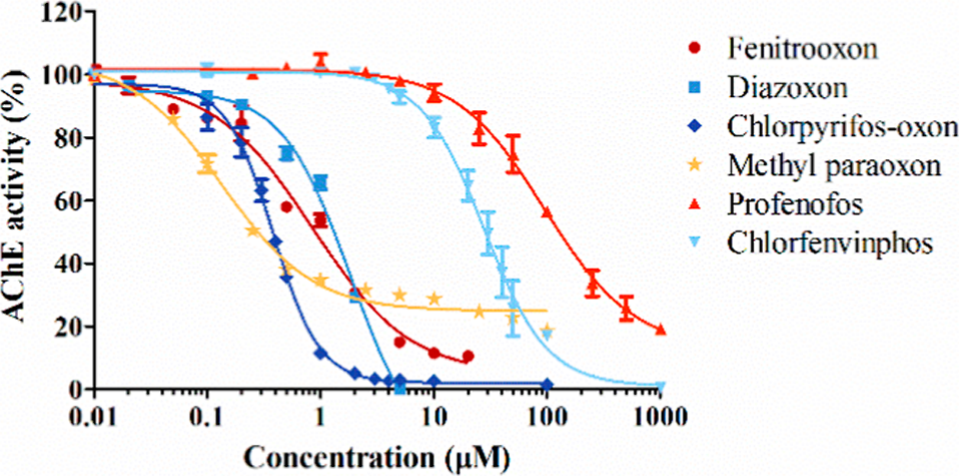
Erythrocyte AChE inhibition determined via in vitro incubations with human blood and fenitrooxon,^27^ diazoxon, chlorpyrifos oxon, methyl paraoxon, profenofos, and chlorfenvinphos. Results are presented as means ± SEM from three independent experiments.

In vitro concentration-based erythrocyte AChE inhibition was determined for the relevant organophosphate oxons (Figure 2). The derived IC_50_ values are 0.14, 0.38, 0.84, 1.82, 27.7, and 94.5 μM for methyl paraoxon, chlorpyrifos oxon, fenitrooxon, diazoxon, chlorfenvinphos, and profenofos, respectively. Additionally, for the comparator compound chlorpyrifos, the derived BMCL_05_ value for its oxon metabolite (chlorpyrifos oxon) is 0.015 μM (detailed results of the BMC analysis are provided in the Supporting Information).

### 3.3 Step 3: Collection of Available Exposure Scenarios and Relevant Adverse Health Effects

Seventy-four human cases reporting single oral exposure to one of the selected OP pesticides were collected, with the corresponding information on the occurrence or absence of adverse health effects (Table S2). Toxic signs and symptoms like weakness, vomiting, headache, dizziness, coma, and even death have been observed in accidental and/or intentional exposure scenarios, where the erythrocyte AChE was substantially inactivated due to the ingestion of large amounts of an OP pesticide.^29−31^ In volunteer studies, abnormal signs and symptoms were usually absent at the relatively low test doses, and erythrocyte AChE activity was monitored as a surrogate end point.^32^ Because of the high sensitivity of erythrocyte AChE, dose levels that resulted in erythrocyte AChE inhibition without concomitant signs and symptoms in the respective participants, as observed, for example, upon a dose of 2 mg/kg BW chlorpyrifos or 1 mg/kg BW chlorfenvinphos,^21,33^ were still considered safe.

Of the collected cases, there were eleven, thirteen, twenty-two, nine, one, and three different dose levels collected for chlorpyrifos, diazinon, fenitrothion, methyl parathion, profenofos, and chlorfenvinphos, respectively (Table 1). Analysis of these data sets indicated that toxic effects were present (positive outcome) for twenty-nine and absent (negative outcome) for twenty-seven of them (Table 1). Meanwhile, three dose levels that had unreported (unknown) health outcomes in humans were also found. Specifically, no information on AChE inhibition and concomitant adverse effects was provided for chlorfenvinphos at 0.18 mg/kg BW, while for diazinon, conflicting opinions on erythrocyte AChE inhibition were reported by the Australian Pesticides and Veterinary Medicines Authority^34^ and the US Environmental Protection Agency^35^ after reviewing the human volunteer study,^36^ of which the description on toxic signs following a single oral DZN administration was also equivocal at dose levels of 0.21 and 0.30 mg/kg BW.

**Table 1:** C*B*_max_oxon Predictions and EAR_test_ and DCR Calculations for the Respective Exposure Levels Collected for Chlorpyrifos, Diazinon, Fenitrothion, Methyl Parathion, Profenofos, and Chlorfenvinphos

exposure level (mg/kg BW)^a^	predicted C*B*_max_oxon (μM)^b^	calculated EAR_test_ value	calculated DCR value	reported or expected adverse health outcome^a^
chlorpyrifos				
0.01	6.8 × 10^–5^	1.8 × 10^–4^	4.6 × 10^–3^	negative
0.012	8.2 × 10^–5^	2.2 × 10^–4^	5.0 × 10^–3^	negative
0.014	9.6 × 10^–5^	2.5 × 10^–4^	6.4 × 10^–3^	negative
0.03	2.1 × 10^–4^	5.4 × 10^–4^	0.014	negative
0.1	6.9 × 10^–4^	1.8 × 10^–3^	0.046	negative
0.5	3.5 × 10^–3^	9.2 × 10^–3^	0.23	negative
1	7.2 × 10^–3^	0.019	0.48	negative
2	0.015	0.040	1.0	negative
214	0.57	1.50	38	positive
286	0.60	1.57	40	positive
300	0.60	1.58	40	positive
diazinon				
0.011	1.5 × 10^–5^	8.3 × 10^–6^	2.1 × 10^–4^	negative
0.03	4.1 × 10^–5^	2.3 × 10^–5^	5.7 × 10^–4^	negative
0.12	1.6 × 10^–4^	9.1 × 10^–5^	2.3 × 10^–3^	negative
0.20	2.8 × 10^–4^	1.5 × 10^–4^	3.8 × 10^–3^	negative
0.21	2.9 × 10^–4^	1.6 × 10^–4^	4.0 × 10^–3^	unknown
0.30	4.1 × 10^–4^	2.3 × 10^–4^	5.8 × 10^–3^	unknown
200	0.15	0.084	2.1	positive
214	0.15	0.085	2.2	positive
293	0.16	0.089	2.3	positive
323	0.16	0.090	2.3	positive
357	0.17	0.091	2.3	positive
429	0.17	0.093	2.3	positive
643	0.17	0.095	2.4	positive
fenitrothion				
0.042	5.7 × 10^–3^	6.8 × 10^–3^	0.17	negative
0.06	8.2 × 10^–3^	9.7 × 10^–3^	0.25	negative
0.083	0.011	0.013	0.34	negative
0.09	0.012	0.015	0.37	negative
0.17	0.023	0.028	0.70	negative
0.18	0.025	0.029	0.74	negative
0.25	0.034	0.041	1.0	negative
0.33	0.045	0.054	1.4	negative
0.36	0.049	0.059	1.5	negative
36	5.79	6.89	174	positive
71	11.47	13.66	346	positive
143	20.24	24.10	610	positive
214	26.90	32.03	811	positive
286	33.34	39.69	1005	positive
357	39.42	46.93	1189	positive
429	45.24	53.86	1364	positive
500	50.60	60.24	1526	positive
536	53.18	63.31	1604	positive
571	55.60	66.18	1677	positive
714	64.64	76.95	1949	positive
1714	100.71	119.90	3037	positive
1786	102.06	121.49	3078	positive
methyl parathion				
0.003	5.1 × 10^–4^	3.6 × 10^–3^	0.092	negative
0.029	4.9 × 10^–3^	0.035	0.89	negative
0.057	9.7 × 10^–3^	0.069	1.8	negative
0.30	0.051	0.36	9.2	negative
26	5.12	36.59	927	positive
171	28.50	203.58	5157	positive
286	40.69	290.61	7362	positive
714	71.03	507.39	12,854	positive
1143	84.26	601.86	15,247	positive
profenofos				
1600	1015.56	10.75	272	positive
chlorfenvinphos				
0.04	4.1 × 10^–3^	1.5 × 10^–4^	3.8 × 10^–3^	negative
0.18	0.019	6.8 × 10^–4^	0.017	unknown
1	0.11	3.9 × 10^–3^	0.098	negative

a See Table S2 for more details and references of original studies.

b Predicted with a generic human PBK model.^22^ The values for fenitrothion and methyl parathion were corrected by dividing the original model predictions by 10 (see Section 2.2.4 for more details).

### 3.4 Step 4: Prediction of Internal Concentrations for Collected Exposure Scenarios

The C*B*_max_oxon for the collected exposure scenarios was predicted for all selected model compounds with a generic human PBK model^22^ and is presented in Table 1. For acute exposure to an organothiophosphate like chlorpyrifos, diazinon, fenitrothion, and methyl parathion, the C*B*_max_oxon was simulated for the corresponding oxon analogue, namely, chlorpyrifos oxon, diazoxon, fenitrooxon, and methyl paraoxon. The original C*B*_max_oxon simulations from the model for dimethyl-organothiophosphates (fenitrothion and methyl parathion) were corrected for the overprediction (see Section 2.2.4), and for the purpose of comparison, the results without correction are presented in Table S3. For a single exposure to an organophosphate oxon (profenofos or chlorfenvinphos), the C*B*_max_oxon was simulated for the parent compound itself.

### 3.5 Step 5: Selection of a Comparator Compound

Chlorpyrifos was chosen as the comparator compound. Toxicity testing following single exposure to this organothiophosphate was investigated with human volunteers, who were fasted overnight and given an oral dose next morning.^21,37^ It was found that 1 mg/kg BW was a dose level where no erythrocyte AChE inhibition took place and that 2 mg/kg BW was the no-observed-adverse-effect level (NOAEL) for toxic signs and symptoms, with a 28% inhibition of erythrocyte AChE reported at this level in one of the participants causing no abnormalities.^21,37^ At the NOAEL of 2 mg/kg BW, the C*B*_max_oxon predicted by the generic human PBK model was 0.015 μM, which was equal to the in vitro derived BMCL_05_ for chlorpyrifos oxon. This indicates that the BMCL_05_ can indeed be considered to represent a safe concentration where no adverse effects occur and hence can be used to define the EAR_comparator_.

### 3.6 Step 6: DCR Calculation for Collected Exposure Scenarios

With the predicted C*B*_max_oxon of the test substances (Table 1) and the in vitro derived IC_50_ values (see Section 3.2), the EAR_test_ values for the collected exposure scenarios were calculated and are listed in Table 1. The EAR_comparator_ was defined with the in vitro derived BMCL_05_ and IC_50_ values for chlorpyrifos oxon, and the resulting value was 0.039. By dividing the EAR_test_ by the EAR_comparator_, a DCR for each exposure scenario was obtained (Table 1).

### 3.7 Step 7: Evaluation and Application of the DCR Approach

**Figure 3:**
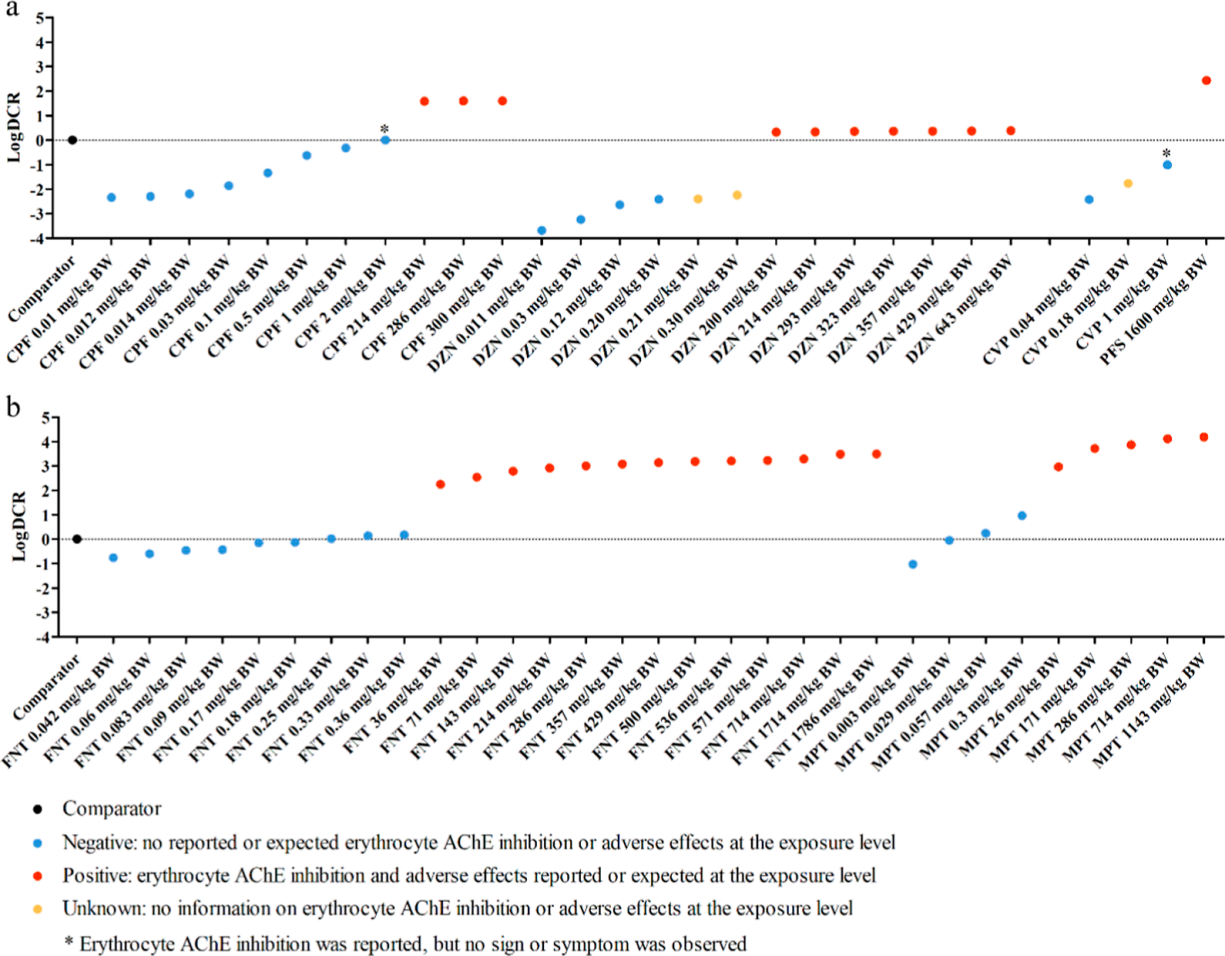
DCR outcomes of collected human cases upon a single oral administration to the selected OP pesticides including (a) chlorpyrifos (CPF), diazinon (DZN), profenofos (PFS), and chlorfenvinphos (CVP) and (b) fenitrothion (FNT) and methyl parathion (MPT). The dotted horizontal lines display the DCR of 1 (LogDCR = 0). Calculated DCR results are shown in Table 1. Details on the evaluation of an exposure scenario as positive, negative, or unknown with respect to the occurrence of erythrocyte AChE inhibition and/or adverse effects are summarized in Table S2.

To evaluate the performance of the obtained DCR-based predictions, we made a comparison between the DCR outcomes and the reported or expected health outcomes for each exposure scenario. Dose levels causing toxic signs and symptoms due to a substantial erythrocyte AChE inactivation are considered to result in positive outcomes, and the DCR approach well-predicted the occurrence of the adverse effects for these exposure scenarios with no false negatives (Figure 3). Also, safe (negative) exposure scenarios inducing no adverse health effects were adequately predicted, except for the false positive results obtained for fenitrothion at 0.33 and 0.36 mg/kg BW and for methyl parathion at 0.057 and 0.30 mg/kg BW, respectively (Figure 3b). These scenarios were considered safe (negative) because no significant erythrocyte AChE inhibition or abnormal signs and symptoms were observed (Table S2). A DCR result close to 1 was obtained for these false positive exposure scenarios (Figure 3b). With a less conservative EAR_comparator_ than the one currently defined based on the BMCL_05_ for erythrocyte AChE inhibition, these false positive scenarios would have been correctly predicted to be negative (DCR ≤ 1); however, this is not preferred because by doing so, the chance of false negative predictions also increases.

Given that no false negatives were obtained, it was concluded that the DCR approach can provide adequate safety evaluation for the acute OP pesticide exposure scenarios by incorporating in vitro derived AChE inhibition potentials with relevant exposure data. The DCR approach was then applied for three exposure scenarios, of which the occurrence of toxic outcomes was unreported (unknown) upon acute exposure to diazinon at 0.21 or 0.30 mg/kg BW or to chlorfenvinphos at 0.18 mg/kg BW. The obtained DCR values for these exposure scenarios are all below 1 (Figure 3a), indicating that these scenarios are predicted to be safe.

## 4 Discussion

In this study, the use of the DCR approach was evaluated as an alternative to animal testing for human health risk assessment of acute OP pesticide exposure. To this end, the EAR_test_ of OP exposure scenarios of interest and the EAR_comparator_ of chlorpyrifos were defined based on toxicity data derived from the in vitro AChE inhibition assay, along with internal exposure data predicted using a newly developed generic human PBK model for OP pesticides.^22^ With the EAR values, the DCR was calculated for the respective exposure scenarios and was then compared with the actual knowledge on the occurrence or absence of in vivo AChE inhibition and adverse effects, enabling the evaluation of the DCR-based predictions.

Four organothiophosphates (chlorpyrifos, diazinon, fenitrothion, and methyl parathion) and two organophosphate oxons (profenofos and chlorfenvinphos) were chosen as model compounds in this work (Figure 1b). All these selected pesticides act as AChE inhibitors after absorption (and metabolic activation to the corresponding oxon analogue) in humans. The inhibition potential of these substances on AChE was determined in vitro with human blood samples (Figure 2), and the erythrocyte AChE inhibition was considered as an adequate surrogate end point for the neuronal AChE inactivation. This surrogate end point is widely adopted in scientific studies^27,38^ and also by regulatory bodies,^39^ given that erythrocyte AChE is more sensitive and easy to sample as compared to the neuronal AChE.^21^ Though the physiological function of erythrocyte AChE is currently unclear, a good correlation has been noted between the severity of clinical signs and symptoms and the degree of erythrocyte AChE inhibition, where approximately 40% remaining erythrocyte AChE activity has been linked with only mild symptoms.^1,31^ For the risk assessment of acute OP pesticide exposure, both 10% and 20% inhibition levels in erythrocyte AChE have been employed by regulatory bodies to define points of departure for setting health-based guidance values.^24,25,40^ In this work, 5% in vitro erythrocyte AChE inhibition was used as a conservative threshold to define the EAR_comparator_. Using a 10% or 20% inhibition would result in a higher EAR_comparator_ and lower DCR predictions and would hence be less protective.

Instead of making comparisons between the predicted C*B*_max_oxon for a given OP exposure and the OP-specific BMCL_05_, the DCR approach that divides an EAR_test_ by a well-defined EAR_comparator_ adds an extra dimension of conservatism to the safety evaluation, since the EAR_test_ is compared to the EAR_comparator_ for a safe exposure level of the comparator compound, taking into account a margin between the predicted blood level and the BMCL_05_ for exposure to the comparator that can be considered safe. Chlorpyrifos was chosen as the comparator compound because compared with other OPs, relevant single exposure studies and NOAELs for humans were well-documented and reviewed by the United States Environmental Protection Agency as a useful assessment for potential erythrocyte AChE inhibition induced by chlorpyrifos.^41^ This enables the validation of the assumption that the in vitro derived BMCL_05_ value resembles a safe internal concentration where no in vivo adverse effect occurs and hence can be used to define an appropriate EAR_comparator_. It is noteworthy that the NOAELs of chlorpyrifos were tested with a limited number of adult participants,^21,37,41^ who may not represent the whole population including susceptible individuals like pregnant women and children. Also, most of the human exposure cases reported (Table S2) and the PBK model used for C*B*_max_oxon predictions^22^ were for adults. Therefore, the EAR and DCR values in the current study are, in the first place, applicable for the general adult population. Their use for susceptible individuals may require the consideration of an uncertainty factor. For example, one could consider adapting the cutoff DCR value from 1 to 0.1 by applying the commonly used uncertainty factor of 10 accounting for human variability in risk assessment. In so doing, confidence would be increased with the negative predictions (DCR ≤ 0.1). However, one could also argue that for a sensitive individual, the EAR_test_ and EAR_comparator_ might be affected to the same extent so that their higher sensitivity can be expected to cancel out when calculating the DCR.

The half-maximum activity concentration, for example, the IC_50_ determined in this study, is considered as the most appropriate metric for EAR_test_ and EAR_comparator_ calculations.^17,18^ This is because the DCR approach (also being referred to as the exposure:activity profiling method) was initially applied for prioritizing chemicals with estrogenic activity.^17^ The estrogenic activity was determined based on various in vitro assays measuring events at different points downstream of receptor binding, and the concentration at half-maximal activity was regarded as the most reliable measurement from different assays to quantify chemical potency.^17^ In this study, the in vitro erythrocyte AChE inhibition of six model OP compounds was tested with the same assay system (human whole blood samples), displaying substrate-specific location and steepness of the concentration–response data (Figure 2). Given that an IC_50_ is the least variable metric and thus provides greater reliability than other metrics along the concentration–response curve,^17^ the IC_50_ values for the model OP compounds were derived from the obtained curves (Figure 2) to determine the inhibition potential and were used as the activity component for calculating EARs (eqs 2 and 3).

The results of the DCR-based safety evaluation reveal that the approach provides adequate predictions for the collected human exposure scenarios of OP pesticides with no false negatives (Figure 3). Still, this approach has uncertainties and limitations, and understanding these is helpful for interpreting the obtained DCR results and making further safety decisions. First, current DCR outcomes might be conservative with regard to the exposure level estimation. The exposure level might be overestimated, especially for accidental and intentional scenarios, where vomiting could happen following OP pesticide poisoning. Besides, the in vitro bioassay (see Section 2.2.2) solely measured the inhibition potential of test OPs on AChE using human whole blood that contains no carboxylesterase,^42^ and the influence of plasma BuChE was also excluded by adding the inhibitor ethopropazine.^12^ In fact, both BuChE and carboxylesterase in the human body (i.e., liver) are able to bind organophosphate oxons,^43−45^ while such protection on AChE is not taken into account in the current DCR calculations. Furthermore, the C*B*_max_oxon was predicted with a generic human PBK model that needs further improvement.^22^ Except for a 10-fold overestimation in C*B*_max_oxon for dimethyl-organothiophosphates (see Section 2.2.4), this deterministic PBK model does not take into account interindividual variations in the (formation and) clearance of organophosphate oxons following administration to OP pesticides.^46−48^ Further refinement of the PBK model by correcting the overestimation and by integrating interindividual variabilities in physiological and toxicokinetic parameters would greatly increase the confidence in the model predictions. Third, the current EAR_comparator_ was built with an in vitro derived BMCL_05_ instead of the predicted C*B*_max_oxon at safe exposure levels. This is based on the consideration of the probable uncertainty in these in vivo dose levels^21,37^ and also because the relevant internal oxon concentration under the safe dose levels was not reported and using a model-predicted C*B*_max_oxon to define the EAR_comparator_ might introduce more uncertainties. Furthermore, using an in vitro derived BMCL_05_ value instead of in vivo data to define the EAR_comparator_ is in line with the principle of the DCR approach as a 3Rs-compliant methodology. It has previously been demonstrated that using a BMCL_05_-defined EAR_comparator_ is adequate in DCR-based safety evaluations for estrogens and antiandrogens,^19,20^ and the results in this work provide a further proof of concept for using an in vitro defined EAR_comparator_ in a DCR-based human safety assessment for acute OP pesticide exposures. Once in silico tools are available for predicting internal concentrations of more OP pesticides, the data sets collected in this work can be extended for a further evaluation on the performance of the current EAR_comparator_.

Of note, the currently collected exposure scenarios are from human volunteer studies and acute poisoning cases (Table S2), for which the exposure dose levels are either very low or high and hence result in explicitly negative or positive DCR outcomes. In the context of environmental exposure to OP pesticides, the OP concentrations commonly measured in human blood samples have been reported to range from 1 × 10^–6^ to 2 × 10^–4^ μM.^49^ Comparing the upper value to the predicted C*B*_max_oxon for six model OPs (Table 1), it indicates that environmental exposure to OP pesticides appears not to raise safety concerns with respect to AChE inactivation and the following acute neurotoxicity. However, AChE inhibition alone cannot account for the adverse effects (i.e., impaired neurobehavioral performance) induced by repeated low-level OP exposures occurring in an environmental context.^50−52^ Although data on internal exposure following repeated exposure can be predicted via computational toxicokinetic modeling, to apply the DCR approach for evaluating the safety from chronic exposure to OP pesticides, information on the underlying mechanism, available in vitro bioassays that can well characterize toxicity, and safe chronic exposure scenarios are required.

To conclude, this study investigated the applicability of the DCR approach for Next Generation Risk Assessment of OP pesticide exposure. Toxicity and internal exposure data used for the EAR and DCR calculations were derived in vitro and in silico. The results suggest that in addition to its application for antiandrogens and estrogens, this DCR-based strategy can also be of use to evaluate the in vivo adverse effects caused by acute exposure to environmental toxicants like OP pesticides, providing another proof of principle for applying this approach for human safety assessment based on the consideration of mode of action, toxicity potency, and exposure context. To further extend the applicability domain of the DCR strategy for more environmental pollutants, available data on the toxic mechanism, toxicity potency, and internal exposure of the compounds of interest as well as an adequate comparator compound with the same mode of action and a known safe exposure level are prerequisites.

## Supporting Information Available

Oral absorption rate constants for all compounds; collected single oral exposure scenarios with reported or expected adverse health effects for all compounds; original C*B*_max_oxon predictions for fenitrothion and methyl parathion; and BMC analysis results for chlorpyrifos oxon (PDF)
