# Supplementary material for: Next Generation Risk Assessment of Acute Neurotoxicity from Organophosphate Exposures Using the In Vitro–In Silico Derived Dietary Comparator Ratio
Source: Environ Sci Technol. 2025 Mar 19;59(12):6106–14. doi: 10.1021/acs.est.5c00220 (PMC11966747; doi:10.1021/acs.est.5c00220)
Supplement: Supplementary file 1 — es5c00220_si_001.pdf [file es5c00220_si_001.pdf]

SUPPORTING INFORMATION

Next Generation Risk Assessment of Acute Neurotoxicity from Organophosphate Exposures Using the *In Vitro-In Silico* Derived Dietary Comparator Ratio

Jiaqi Chen[a,], Thijs M.J.A. Moerenhout[a], Nynke I. Kramer[a], Ivonne M.C.M. Rietjens[a]*

[a] Division of Toxicology, Wageningen University and Research, Stippeneng 4, 6708 WE, Wageningen, The Netherlands

* Corresponding author: Jiaqi Chen (jiaqi.chen@wur.nl)

17 pages, 3 Tables

Table S1 Quantitative structure–activity relationship (QSAR)-based predictions of oral absorption rate constants for chlorpyrifos, diazinon, fenitrothion, methyl parathion, profenofos, and chlorfenvinphos.

**Compound**	**Oral absorption rate constant (hr^{-1})**	**Reference**
Chlorpyrifos	1.01	Punt et al. 2022;
Diazinon	0.73	Moerenhout et al. 2024
Fenitrothion	0.58	
Methyl parathion	0.58	
Profenofos	0.90	
Chlorfenvinphos	0.81	

Table S2 Summary of collected human cases with reported or expected adverse health effects following single oral exposure to chlorpyrifos, diazinon, fenitrothion, methyl parathion, profenofos or chlorfenvinphos.

Human cases	Exposure dose levels (mg/kg BW) [a]	Reported AChE inhibition and concomitant effects	Reference	Expected AChE inhibition and concomitant effects	Reasoning
**Chlorpyrifos**					
Single oral exposure in 4 male volunteers	0.01	No erythrocyte AChE inhibition was observed	van Gemert et al. 2001 (original data of Coulston et al. 1972)	No	No erythrocyte AChE inhibition was reported
Single oral exposure of 1 mg (2852 nmol) in 4 male and 1 female volunteers (age 26 - 45 years, BW 73 - 92 kg), dose was given in a sugar cube	0.012 (calculated with a mean BW of 82.5 kg)	Erythrocyte AChE activity did not fall below 90% of that before exposure during dosing regime	Griffin et al. 1999	No	No significant erythrocyte AChE inhibition was reported
Single oral exposure in 4 male volunteers	0.014 [b]	No erythrocyte AChE inhibition was observed	van Gemert et al. 2001 (original data of Coulston et al. 1972)	No	No erythrocyte AChE inhibition was reported
Single oral exposure in 4 male volunteers	0.03	No erythrocyte AChE inhibition was observed	van Gemert et al. 2001 (original data of Coulston et al. 1972)	No	No erythrocyte AChE inhibition was reported
Single oral exposure in 4 male volunteers	0.1	No erythrocyte AChE inhibition was observed	van Gemert et al. 2001 (original data of Coulston et al. 1972)	No	No erythrocyte AChE inhibition was reported
Single oral exposure in 6 male volunteers (age 27 - 50 years, mean BW 83.3 kg), dose was given in a tablet with 100 mL water after a breakfast	0.5	No change in erythrocyte AChE activity was observed	Nolan et al. 1984	No	No erythrocyte AChE inhibition was reported
Single oral exposure in capsule form in 6 male and 6 female volunteers (age 19 - 54 years, BW 44 - 102 kg, fasted overnight)	0.5	No statistical differences in mean erythrocyte AChE activity were identified in volunteers	van Gemert et al. 2001, US EPA. 2009 (original data of Kisicki et al. 1999)	No	No significant erythrocyte AChE inhibition was reported

Human cases	Exposure dose levels (mg/kg BW) [a]	Reported AChE inhibition and concomitant effects	Reference	Expected AChE inhibition and concomitant effects	Reasoning
Single oral exposure in capsule form in 6 male and 6 female volunteers (age 19 - 54 years, BW 44 - 102 kg, fasted overnight)	1	No statistical differences in mean erythrocyte AChE activity were identified in volunteers	van Gemert et al. 2001, US EPA. 2009 (original data of Kisicki et al. 1999)	No	No erythrocyte AChE inhibition was reported
Single oral exposure in capsule form in 6 male and 6 female volunteers (age 19 - 54 years, BW 44 - 102 kg, fasted overnight)	2	One female volunteer had a decreased erythrocyte AChE activity (72%), but no abnormal signs and symptoms was observed	van Gemert et al. 2001, US EPA. 2009 (original data of Kisicki et al. 1999)	No	Erythrocyte AChE inhibition (28%) was found, but no concomitant signs and symptoms were observed
Acute oral exposure of 15 - 30 g (30 - 60 mL of a 50% formulation) in a 25-year-old male	214 (estimated with the minimum dose level)	Significantly decreased erythrocyte AChE activity, and toxic symptoms occurred	Drevenkar et al. 1993	Yes	Significant erythrocyte AChE inhibition was found, and toxic symptoms occurred
Acute oral exposure of 15 - 30 g (30 - 60 mL of a 50% formulation) in a 28-year-old female	214 (estimated with the minimum dose level)	Significantly decreased erythrocyte AChE activity, and toxic symptoms occurred	Drevenkar et al. 1993	Yes	Significant erythrocyte AChE inhibition was found, and toxic symptoms occurred
Acute oral exposure of 20 g in a 48-year-old male	286	Toxic symptoms occurred, no information reported on erythrocyte AChE inhibition	Martinez-Chuecos et al. 1992	Yes	Toxic symptoms occurred
Acute oral exposure in a 42-year-old male	300	Significantly decreased erythrocyte AChE inhibition, and toxic symptoms occurred	Lotti et al. 1986	Yes	Significant erythrocyte AChE inhibition was found, and toxic symptoms occurred
**Diazinon**					
Single oral exposure in 4 male and 1 female volunteers (age 30 - 50 years, BW 76 - 90 kg), dose was given in a diluted ethanolic solution with 200 mL water	0.011	No erythrocyte AChE inhibition was observed	Garfitt et al. 2002	No	No erythrocyte AChE inhibition was reported

Human cases	Exposure dose levels (mg/kg BW) [a]	Reported AChE inhibition and concomitant effects	Reference	Expected AChE inhibition and concomitant effects	Reasoning
Single oral exposure in 7 male volunteers (age 18 - 48 years, BW 73.7 - 89.0 kg), dose was given in gelatine capsules with 240 mL water after consuming a standardized breakfast	0.03	No erythrocyte AChE inhibition was observed	APVMA. 2011;<br>US EPA. 2001 (original data of Boyeson. 2000)	No	No erythrocyte AChE inhibition was reported
Single oral exposure in 7 male volunteers (age 18 - 48 years, BW 62.2 - 98.6 kg), dose was given in gelatine capsules with 240 mL water after consuming a standardized breakfast	0.12	No erythrocyte AChE inhibition was observed	APVMA. 2011;<br>US EPA. 2001 (original data of Boyeson. 2000)	No	No erythrocyte AChE inhibition was reported
Single oral exposure in 7 male volunteers (age 18 - 48 years, BW 71.6 - 88.7 kg), dose was given in gelatine capsules with 240 mL water after consuming a standardized breakfast	0.20	No erythrocyte AChE inhibition was observed	APVMA. 2011;<br>US EPA. 2001 (original data of Boyeson. 2000)	No	No erythrocyte AChE inhibition was reported
Single oral exposure in 7 male volunteers (age 18 - 48 years, BW 66.4 - 95.2 kg), dose was given in gelatine capsules with 240 mL water after consuming a standardized breakfast	0.21	**APVMA**: significant erythrocyte AChE inhibition at this dose level; **US EPA**: limited significant erythrocyte AChE inhibition at this dose level, and did not follow the inhibition pattern.<br>Mild clinical signs or symptoms might occur	APVMA. 2011;<br>US EPA. 2001 (original data of Boyeson. 2000)	Unknown	Conflicting opinions on erythrocyte AChE activity data, mild clinical signs or symptoms might occur

Human cases	Exposure dose levels (mg/kg BW) [a]	Reported AChE inhibition and concomitant effects	Reference	Expected AChE inhibition and concomitant effects	Reasoning
Single oral exposure in 1 male adult volunteer (age unknown, BW 71.2 kg), dose was given in gelatine capsules with 240 mL water after consuming a standardized breakfast	0.30	**APVMA**: though with an inconsistent inhibition pattern, erythrocyte AChE inhibition was greater than that observed at 0.20 mg/kg BW, and was persistent from day one post-dose onwards; **US EPA**: no erythrocyte AChE inhibition at this dose level. Mild clinical signs or symptoms might occur	APVMA. 2011; US EPA. 2001 (original data of Boyeson. 2000)	Unknown	Conflicting opinions on erythrocyte AChE activity data, mild clinical signs or symptoms might occur
Acute oral exposure of 14 - 57 g (presumably concentrate formulation) in 14 males and 11 females (age 1.5 - 60 years)	200 (estimated with the minimum dose level)	Toxic symptoms occurred, no information reported on erythrocyte AChE inhibition	APVMA. 2011 (original data of Mutalik et al. 1962)	Yes	Toxic symptoms occurred
Acute oral exposure of 15 g (60 mL of a 25% formulation) in a 21-year-old male	214	Significantly decreased erythrocyte AChE activity, and toxic symptoms occurred	Klemmer et al. 1978	Yes	Significant erythrocyte AChE inhibition was found, and toxic symptoms occurred
Acute oral exposure of 22 g in a 54-year-old female (BW 75 kg)	293	Died, no information reported on erythrocyte AChE inhibition	Poklis et al. 1980	Yes	Toxic symptoms (death) occurred
Acute oral exposure of 22.6 g (113 g of a 20% formulation) in a 19-year-old male	323	Toxic symptoms occurred, no information reported on erythrocyte AChE inhibition	APVMA. 2011 (original data of Banerjee. 1967)	Yes	Toxic symptoms occurred
Acute oral exposure of 25 g (100 mL of a 25% formulation) in a 20-year-old female	357	Significantly decreased erythrocyte AChE inhibition, and toxic symptoms occurred	Klemmer et al. 1978	Yes	Significant erythrocyte AChE inhibition was found, and toxic symptoms occurred
Acute oral exposure of 25 g (100 mL of a 25% formulation) in a 49-year-old male	357	Significantly decreased erythrocyte AChE inhibition, and toxic symptoms occurred	Klemmer et al. 1978	Yes	Significant erythrocyte AChE inhibition was found, and toxic symptoms occurred

Human cases	Exposure dose levels (mg/kg BW) [a]	Reported AChE inhibition and concomitant effects	Reference	Expected AChE inhibition and concomitant effects	Reasoning
Acute oral exposure of 30 g in a 48-year-old male	429	Toxic symptoms occurred, no information reported on erythrocyte AChE inhibition	Martinez-Chuecos et al. 1992	Yes	Toxic symptoms occurred
Acute oral exposure of 45 g (180 mL of a 25% formulation) in a 19-year-old male	643	Significantly decreased erythrocyte AChE inhibition, and toxic symptoms occurred	Klemmer et al. 1978	Yes	Significant erythrocyte AChE inhibition was found, and toxic symptoms occurred
**Fenitrothion**					
Single oral exposure in 3 volunteers, dose was given in gelatine capsules in the morning	0.042	No significant erythrocyte AChE inhibition was observed	Nosáľ and Hladká. 1968	No	No significant erythrocyte AChE inhibition was reported
Single oral exposure in 3 male volunteers (mean age 45 years)	0.06	No significant symptoms or adverse effects were observed, no information reported on erythrocyte AChE inhibition	Meaklim et al. 2003	No	No significant symptoms or adverse effects were observed
Single oral exposure in 9 volunteers, dose was given in gelatine capsules in the morning	0.083	No significant erythrocyte AChE inhibition was observed	Nosáľ and Hladká. 1968	No	No significant erythrocyte AChE inhibition was reported
Single oral exposure in 8 male and 4 female volunteers (mean age 33 years), dose was given in capsule and taken with food	0.09 [b]	No significant erythrocyte AChE inhibition was observed	Meaklim et al. 2003	No	No significant erythrocyte AChE inhibition was reported
Single oral exposure in 5 volunteers, dose was given in gelatine capsules in the morning	0.17	No significant erythrocyte AChE inhibition was observed	Nosáľ and Hladká. 1968	No	No significant erythrocyte AChE inhibition was reported
Single oral exposure in 3 male volunteers (mean age 45 years)	0.18	No significant symptoms or adverse effects was observed, no information reported on erythrocyte AChE inhibition	Meaklim et al. 2003	No	No significant symptoms or adverse effects was observed

Human cases	Exposure dose levels (mg/kg BW) [a]	Reported AChE inhibition and concomitant effects	Reference	Expected AChE inhibition and concomitant effects	Reasoning
Single oral exposure in 8 male and 4 female volunteers (mean age 33 years), dose was given in capsule and taken with food	0.18 [b]	No significant erythrocyte AChE inhibition was observed	Meaklim et al. 2003	No	No significant erythrocyte AChE inhibition was reported
Single oral exposure in 2 volunteers, dose was given in gelatine capsules in the morning	0.25	No significant erythrocyte AChE inhibition was observed	Nosáľ and Hladká. 1968	No	No significant erythrocyte AChE inhibition was reported
Single oral exposure in 5 volunteers, dose was given in gelatine capsules in the morning	0.33	No significant erythrocyte AChE inhibition was observed	Nosáľ and Hladká. 1968	No	No significant erythrocyte AChE inhibition was reported
Single oral exposure in 3 male volunteers (mean age 45 years)	0.36	No symptoms or adverse effects was observed, no information reported on erythrocyte AChE inhibition	Meaklim et al. 2003	No	No symptoms or adverse effects was observed
Acute oral exposure of 2.5 g (50 mL of a 5% formulation) in a 23-year-old male	36	Toxic symptoms occurred, no information reported on erythrocyte AChE inhibition	Kojima et al. 1989	Yes	Toxic symptoms occurred
Acute oral exposure of 5 g in a 28-year-old female	71	Toxic symptoms occurred, no information reported on erythrocyte AChE inhibition	Yoshida and Kuroki. 2010 (original data of Koyama et al. 2006)	Yes	Toxic symptoms occurred
Acute oral exposure of 10 g in a 76-year-old female	143	Toxic symptoms occurred, no information reported on AChE inhibition	Yoshida and Kuroki. 2010 (original data of Koyama et al. 2006)	Yes	Toxic symptoms occurred
Acute oral exposure of 15 g in a 79-year-old female	214	Toxic symptoms occurred, no information reported on erythrocyte AChE inhibition	Yoshida and Kuroki. 2010 (original data of Koyama et al. 2006)	Yes	Toxic symptoms occurred
Acute oral exposure of 15 g in a 33-year-old male	214	Toxic symptoms occurred, no information reported on erythrocyte AChE inhibition	Yoshida and Kuroki. 2010 (original data of Koyama et al. 2006)	Yes	Toxic symptoms occurred

Human cases	Exposure dose levels (mg/kg BW) [a]	Reported AChE inhibition and concomitant effects	Reference	Expected AChE inhibition and concomitant effects	Reasoning
Acute oral exposure of 20 g (40 mL of a 50% formulation) in a 70-year-old female	286	Toxic symptoms occurred, no information reported on erythrocyte AChE inhibition	Sakamoto et al. 1984	Yes	Toxic symptoms occurred
Acute oral exposure of 25 g in a 24-year-old female	357	Toxic symptoms occurred, no information reported on erythrocyte AChE inhibition	Yoshida and Kuroki. 2010 (original data of Koyama et al. 2006)	Yes	Toxic symptoms occurred
Acute oral exposure of 25 g in a 65-year-old male	357	Toxic symptoms occurred, no information reported on erythrocyte AChE inhibition	Yoshida and Kuroki. 2010 (original data of Koyama et al. 2006)	Yes	Toxic symptoms occurred
Acute oral exposure of 25 g in a 48-year-old male	357	Toxic symptoms occurred, no information reported on erythrocyte AChE inhibition	Martinez-Chuecos et al. 1992	Yes	Toxic symptoms occurred
Acute oral exposure of 30 g (60 mL of a 50% formulation) in a 56-year-old male	429	Toxic symptoms occurred, no information reported on erythrocyte AChE inhibition	Yoshida et al. 1987	Yes	Toxic symptoms occurred
Acute oral exposure of 30.0 ± 19.7 g in 4 males and 6 females (mean age 62.5 ± 11.9 years)	429 (estimated with the mean dose level)	Toxic symptoms occurred, no information reported on erythrocyte AChE inhibition	Inoue et al. 2008	Yes	Toxic symptoms occurred
Acute oral exposure of 35 g in a 73-year-old male	500	Toxic symptoms occurred, no information reported on erythrocyte AChE inhibition	Yoshida and Kuroki. 2010 (original data of Koyama et al. 2006)	Yes	Toxic symptoms occurred
Acute oral exposure of 35 g in a 47-year-old female	500	Toxic symptoms occurred, no information reported on erythrocyte AChE inhibition	Yoshida and Kuroki. 2010 (original data of Koyama et al. 2006)	Yes	Toxic symptoms occurred
Acute oral exposure of 37.5 g in a 39-year-old male	536	Toxic symptoms occurred, no information reported on erythrocyte AChE inhibition	Yoshida and Kuroki. 2010 (original data of Koyama et al. 2006)	Yes	Toxic symptoms occurred
Acute oral exposure of 40 g (100 mL of a 40% formulation) in a 60-year-old female	571	Toxic symptoms occurred, no information reported on erythrocyte AChE inhibition	Park and Choi. 2017	Yes	Toxic symptoms occurred

Human cases	Exposure dose levels (mg/kg BW) [a]	Reported AChE inhibition and concomitant effects	Reference	Expected AChE inhibition and concomitant effects	Reasoning
Acute oral exposure of 50 g in a 50-year-old female	714	Toxic symptoms occurred, no information reported on erythrocyte AChE inhibition	Yoshida and Kuroki. 2010 (original data of Koyama et al. 2006)	Yes	Toxic symptoms occurred
Acute oral exposure of 50 g in a 57-year-old female	714	Toxic symptoms occurred, no information reported on erythrocyte AChE inhibition	Yoshida and Kuroki. 2010 (original data of Koyama et al. 2006)	Yes	Toxic symptoms occurred
Acute oral exposure of 50 g in a 60-year-old male	714	Toxic symptoms occurred, no information reported on erythrocyte AChE inhibition	Yoshida and Kuroki. 2010 (original data of Koyama et al. 2006)	Yes	Toxic symptoms occurred
Acute oral exposure of 50 g in a 62-year-old female	714	Toxic symptoms occurred, no information reported on erythrocyte AChE inhibition	Yoshida and Kuroki. 2010 (original data of Koyama et al. 2006)	Yes	Toxic symptoms occurred
Acute oral exposure of 50 g in a 35-year-old female	714	Toxic symptoms occurred, no information reported on erythrocyte AChE inhibition	Yoshida and Kuroki. 2010 (original data of Koyama et al. 2006)	Yes	Toxic symptoms occurred
Acute oral exposure of 50 g in a 51-year-old male	714	Toxic symptoms occurred, no information reported on erythrocyte AChE inhibition	Yoshida and Kuroki. 2010 (original data of Koyama et al. 2006)	Yes	Toxic symptoms occurred
Acute oral exposure of 120 g in 13 males and 2 females (mean age 65 years)	1714	Significantly decreased erythrocyte AChE activity, and toxic symptoms occurred	Moon et al. 2015	Yes	Significant erythrocyte AChE inhibition was found with concomitant toxic symptoms
Acute oral exposure of 125.0 ± 35.4 g in one male and one female (mean age 56.5 ± 0.7 years)	1786 (estimated with the mean dose level)	Toxic symptoms occurred, no information reported on erythrocyte AChE inhibition	Inoue et al. 2008	Yes	Toxic symptoms occurred
**Methyl parathion**					
Single oral exposure in 6 male volunteers (age 18 - 45 years) (fasted overnight), dose was given in a diluted ethanolic solution	0.003	No information reported on erythrocyte AChE activity and toxic signs and symptoms	APVMA. 2011 (original data of Freestone et al. 2002)	No	This dose level was the acceptable daily intake (ADI) for humans (APVMA. 2011)

Human cases	Exposure dose levels (mg/kg BW) [a]	Reported AChE inhibition and concomitant effects	Reference	Expected AChE inhibition and concomitant effects	Reasoning
Single oral exposure of 2 mg in 2 male volunteers (53 and 62 year-old), dose was given corn oil dispensed onto a cookie	0.029 [b]	No significant erythrocyte AChE inhibition was observed, and no adverse effects was observed	Rodnitzky et al. 1978	No	No significant erythrocyte AChE inhibition and concomitant adverse effects was reported
Single oral exposure of 4 mg in 2 male volunteers (53 and 62 year-old), dose was given corn oil dispensed onto a cookie	0.057 [b]	No significant erythrocyte AChE inhibition was observed, and no adverse effects was observed	Rodnitzky et al. 1978	No	No significant erythrocyte AChE inhibition and concomitant adverse effects was reported
Single oral exposure of 20 mg in 5 volunteers	0.3 [b]	No significant erythrocyte AChE inhibition was observed	APVMA. 1999 (original data of Rider and Puletti. 1971)	No	No significant erythrocyte AChE inhibition was reported
Acute oral exposure of in a 50-year-old male	26	Died, no information reported on erythrocyte AChE inhibition	Fazekas and Rengei. 1965	Yes	Toxic symptoms (death) occurred
Acute oral exposure of 12 - 24 g (50 - 100 mL of 240 g/L formulation) in a 29-year-old male	171	Significantly decreased erythrocyte AChE activity, toxic signs occurred	Isbister et al. 2007	Yes	Significant erythrocyte AChE inhibition was found with concomitant toxic signs
Acute oral exposure of 20 g (100 mL of a 20% formulation) in a 70-year-old male	286	Significantly decreased blood ChE activity (unclear if it is erythrocyte AChE or plasma BuChE); died	Luzhnikov et al. 1977	Yes	Toxic symptoms (death) occurred
Acute oral exposure of 20 g (100 mL of a 20% formulation) in a 17-year-old male	286	Significantly decreased blood ChE activity (unclear if it is erythrocyte AChE or plasma BuChE); no information reported on clinical details	Luzhnikov et al. 1977	Yes	Toxic symptoms were expected to take place following the large ingestion (reported blood concentration was 3 mg/L)
Acute oral exposure of 50 -300 g in 26 subjects	714 (estimated with the minimum dose level)	Died, no information reported on erythrocyte AChE inhibition	Fazekas. 1971	Yes	Toxic symptoms (death) occurred
Acute oral exposure of 80 g (400 mL of a 20% formulation) in a 25-year-old male	1143	Significantly decreased erythrocyte AChE activity, toxic symptoms occurred	Luzhnikov et al. 1977	Yes	Significant erythrocyte AChE inhibition was found with concomitant symptoms

Human cases	Exposure dose levels (mg/kg BW) [a]	Reported AChE inhibition and concomitant effects	Reference	Expected AChE inhibition and concomitant effects	Reasoning
**Profenofos**					
Acute oral exposure of 112 g (280 mL of a 40% formulation) in a 88-year-old female	1600	Died, no information reported on erythrocyte AChE inhibition	Seno et al. 1998	Yes	Toxic symptoms (death) occurred
**Chlorfenvinphos**					
Single oral exposure of 3 mg in male volunteers	0.04 [b]	No information reported on erythrocyte AChE inhibition, no toxic signs was observed	APVMA. 2000 (original data of Hunter et al. 1972)	No	No toxic signs was observed
Single oral exposure of 12.5 mg in a male volunteer, dose was given in 0.75 mL olive oil	0.18	No information reported on erythrocyte AChE inhibition and concomitant signs and symptoms	APVMA. 2000 (original data of Hutson. 1969)	Unknown	No information reported on erythrocyte AChE inhibition and concomitant signs and symptoms
Single oral exposure in a male volunteer (age 35 - 40 years), dose was given in a gelatine capsule	1	Decreased erythrocyte AChE activity, but no toxic signs and symptoms was observed	APVMA. 2000 (original data of Brown. 1966)	No	Erythrocyte AChE inhibition was found (40%), but no toxic signs and symptoms was observed

a: reported data, or data estimated with the available ingested amount (in mg) and a body weight of 70 kg regardless of age, sex and ethnicity, unless a specific body weight was provided;
b: repeated exposure study in human volunteers, the scenario following the first oral administration was taken as the single exposure scenario.

Table S3 Original CB_{max}oxon predictions for the collected exposure dose levels of fenitrothion and methyl parathion.

Exposure level (mg/kg BW) [a]	Predicted CB_{max}oxon (μM) without correction [b]	Exposure level (mg/kg BW) [a]	Predicted CB_{max}oxon (μM) without correction [b]
**Fenitrothion**		**Methyl parathion**	
0.042	0.057	0.003	5.1×10^{-3}
0.06	0.082	0.029	0.049
0.083	0.11	0.057	0.097
0.09	0.12	0.30	0.51
0.17	0.23	26	51.23
0.18	0.25	171	285.01
0.25	0.34	286	406.86
0.33	0.45	714	710.34
0.36	0.49	1143	842.60
36	57.86		
71	114.71		
143	202.42		
214	269.04		
286	333.38		
357	394.23		
429	452.41		
500	506.01		
536	531.78		
571	555.95		
714	646.38		
1714	1007.14		
1786	1020.55		

a: See Table S2 for more details and references of original studies;
b: See section 2.2.4 for more details.

BMC analysis for chlorpyrifos-oxon

The EFSA web-tool (https://efsa.openanalytics.eu/) integrated with the R package PROAST (version 70.0) was employed for the BMC analysis. Briefly, the continuous data (the obtained erythrocyte AChE activity in human blood upon *in vitro* incubations with increasing concentrations of chlorpyrifos-oxon) were fitted to a set of models (Exponential, Hill, Inverse Exponential, and Log-Normal family models), and all fitted models excluding the FULL and NULL models were used for model averaging via a weighted average model. More weight was given to the models with lower Akaike's Information Criterion (AIC), and an averaged confidence interval was estimated using the recommended defaults. The table and figures present the characteristics of fitted models, the weights for model averaging, and the benchmark concentration for 5% erythrocyte AChE inhibition with the 95% lower–upper confidence limits (BMCL–BMCU).

Model	Converged	loglik	npar	AIC	Weights	Final BMC values (µM)	
full	NA	NA	NA	NA	-	$BMCL_{05}$	$BMCU_{05}$
null model	yes	-70.36	2	144.72	-	0.015	0.10
Expon. m3-	yes	-46.83	4	101.66	-		
Expon. m5-	yes	-14.32	5	38.64	0.05		
Hill m3-	yes	-45.81	4	99.62	-		
Hill m5-	yes	-11.78	5	33.56	0.60		
Inv.Expon. m3-	yes	-37.99	4	83.98	-		
Inv.Expon. m5-	yes	-19.42	5	48.84	0		
LN m3-	yes	-40.79	4	89.58	-		
LN m5-	yes	-12.31	5	34.62	0.35		

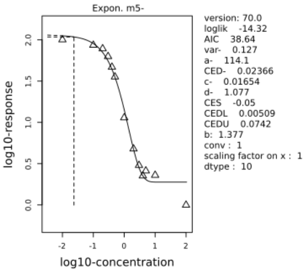

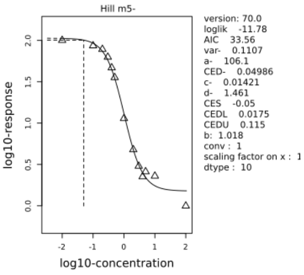

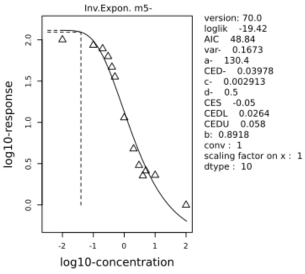

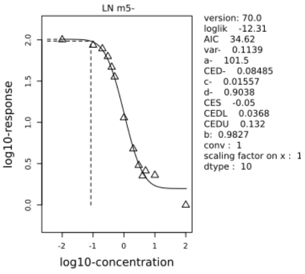

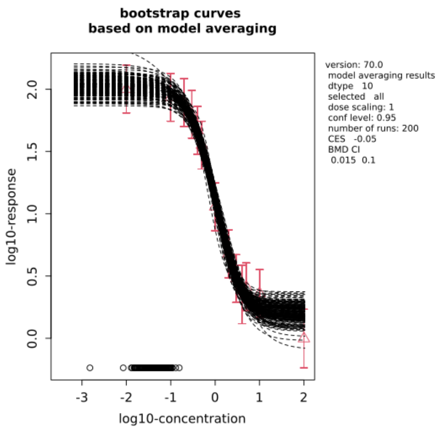

References

APVMA (1999). Parathion-methyl interim review report: Toxicology assessment. https://webarchive.nla.gov.au/awa/20150622210408/http://apvma.gov.au/node/14791 (accessed 16 May 2024).

APVMA (2000). Chlorfenvinphos interim review report: Toxicology assessment. https://webarchive.nla.gov.au/awa/20150622200808/http://apvma.gov.au/node/14716 (accessed 16 May 2024).

APVMA (2011). Diazinon: Human health risk assessment, Part 2: Toxicological hazard assessment. https://webarchive.nla.gov.au/awa/20151020211805/http://apvma.gov.au/node/18711 (accessed 19 April 2024).

APVMA (2011). Parathion-methyl final review report: The reconsideration of the active constituent parathion-methyl, registration of products containing parathion-methyl and approvals of their associated labels. https://webarchive.nla.gov.au/awa/20151020220414/http://apvma.gov.au/node/14806 (accessed 16 May 2024).

Banerjee D (1967). Pericarditis in acute diazinon poisoning. Armed Forces Medical College, Poona, India. Armed Forces Med J (India) 23: 187-190.

Boyeson MG (2000). A randomized, double-blind, ascending, acute, oral dose study of diazinon to determine the no effect level (NOEL) for plasma and RBC cholinesterase activity in normal, healthy subjects (study no. NCP-8373, Part A); Unpublished report (Novartis no. 587-98); Covance Clinical Research Unit Inc.: Madison, WI. https://webarchive.nla.gov.au/awa/20151020211805/http://apvma.gov.au/node/18711 (accessed 19 April 2024).

Brown VKH (1966). The effect of chlorfenvinphos (SD 7859) on two human volunteers. IRR TL/18/66. Shell Research Institute, Sittingbourne [Sh sub no 1382 A3162/1 Box 82, A3162/1Box 38].

Coulston F, Golberg L, Griffin T (1972). Safety Evaluation of Dowco 179 in Human Volunteers. Unpublished report from the Institute of Experimental Pathology and Toxicology, Albany Medical College.

Drevenkar V, Vasilić Z, Stengl B, Fröbe Z, Rumenjak V (1993). Chlorpyrifos metabolites in serum and urine of poisoned persons. Chemico-Biological Interactions 87:315-322.

Fazekas IG (1971). Über die makroskopischen und mikroskopischen Veränderungen bei der Wofatox-Vergiftung (Methyl-Parathion). Z Rechtsmed 68:189-194.

Fazekas IG, Rengei B (1965). Tödliche Vergiftung mit Methyl-Parathion ("Wofatox"). Arch. Toxikol. 20:323-326.

Freestone S, Lindemann JPO, Growcott IS (2002). A human metabolism study to determine elimination of p-nitrophenol and conjugates after a single oral administration of methyl-parathion. Inveresk Report Number 20442 (Study No 5962).

Garfitt SJ, Jones K, Mason HJ, Cocker J (2002). Exposure to the organophosphate diazinon: data from a human volunteer study with oral and dermal doses. Toxicology Letters 134:105-113.

Griffin P, Mason H, Heywood K, Cocker J (1999). Oral and dermal absorption of chlorpyrifos: a human volunteer study. Occupational and Environmental Medicine 56(1):10-13.

Hunter CG, Robinson J, Bedford CT, Lawson JM (1972). Exposure to chlorfenvinphos by determination of a urinary metabolite. J Occup Med 14:119-122.

Hutson D (1969). The metabolism of [^{14}C] chlorfenvinphos in man. TLGR.0006.69, Shell Research Institute, Sittingbourne [Sh sub no 1385 A3162/1 Box 82, A3162/5 Box 39].

Inoue S, Saito T, Suzuki Y, Iizuka S, Takazawa K, Akieda K, Yamamoto I, Inokuchi S (2008). Prognostic factors and toxicokinetics in acute fenitrothion self-poisoning requiring intensive care. Clinical Toxicology 46(6):528-533.

Isbister GK, Mills K, Friberg LE, Hodge M, O'Connor E, Patel R, Abeyewardene M, Eddleston M (2007). Human methyl parathion poisoning. Clinical Toxicology 45(8):956-960.

Kisicki JC, Seip CW, Combs ML (1999). A Rising Dose Toxicology Study to Determine the No-Observable-Effect-Levels (NOEL) for Erythrocyte Acetylcholinesterase (AChE) Inhibition and Cholinergic Signs and Symptoms of Chlorpyrifos at Three Dose Levels, Unpublished report from M. D. S. Harris.

Klemmer HW, Reichert ER, Yauger WL, Haley TJ (1978). Five Cases of Intentional Ingestion of 25 Percent Diazinon with Treatment and Recovery. Clinical Toxicology 12(4):435-444.

Kojima T, Yashiki M, Miyazaki T, Chikasue F, Ohtani M (1989). Detection of S-methylfenitrothion, aminofenitrothion, aminofenitroxon and acetylaminofenitroxon in the urine of a fenitrothion intoxication case. Forensic Science International 41(3):245-253.

Koyama K, Suzuki R, Kikuno T, Kaziwara H, and Shinba T (2006). Serum fenitrothion concentration and toxic symptom in acute intoxication patients. Jpn J. Toxicol. 19:41-47.

Lotti M, Moretto A, Zoppellari R, Dainese R, Rizzuto N, Barusco G (1986). Inhibition of lymphocytic neuropathy target esterase predicts the development of organophosphate-induced delayed polyneuropathy. Archives of Toxicology 59:176–179.

Luzhnikov EA, Yaroslavsky AA, Molodenkov MN, Shurkalin BK, Evseev NG, Barsukov UF (1977). Lancet 310(8027):38-39.

Martinez-Chuecos J, Carmen Jurado MD, Gimenez MP, Martinez D, Menendez M (1992). Experience with hemoperfusion for organophosphate poisoning. Critical Care Medicine 20(11):1538-1543.

Meaklim J, Yang J, Drummer OH, Killalea S, Staikos V, Horomidis S, Rutherford D, Ioannides-Demos LL, Lim S, McLean AJ, McNeil JJ (2003). Fenitrothion: Toxicokinetics and toxicologic evaluation in human volunteers. Environmental Health Perspectives 111:305-308.

Moerenhout, TMJA, Chen J, Bouwmeester H, Rietjens IMCM, Kramer NI (2024). Development of a generic physiologically based kinetic model for the prediction of internal exposure to organophosphate pesticides. Environ. Sci. Technol 58(42):18834-18845.

Moon J, Chun B, Lee S (2015). Variable response of cholinesterase activities following human exposure to different types of organophosphates. Human & Experimental Toxicology 34(7):698-706.

Mutalik GS, Wadia RS, Pai VR (1962). Poisoning by diazinon, an organophosphate insecticide. Dept of Medicine, B.J. Medical College, Poona, India. J Indian Med Assoc 38: 67-71.

Nolan RJ, Rick DL, Freshour NL, Saunders JH (1984). Chlorpyrifos: Pharmacokinetics in human volunteers. Toxicology and Applied Pharmacology 73(1):8-15.

Nosáľ M, Hladká A (1968). Determination of the exposure to fenitrothion (O,O-dimethyl-O/3-methyl-4-nitrophenyl/thiophosphate) on the basis of the excretion of p-nitro-m-cresol by the urine of the persons tested. Int Arch Gewerbepath Gewerbehyg 25:28-38.

Park JT, Choi KH (2017). Polyneuropathy following acute fenitrothion poisoning. Clinical Toxicology 56(5):385-386.

Poklis A, Kutz FW, Sperling JF, Morgan DP (1980). A fatal diazinon poisoning. Forensic Science International 15(2):135-140.

Rider SJI, Puletti EJ (1971). Anticholinesterase toxicity studies with methyl parathion, guthion and phosdrin in human subjects. Fed. Proc. 30(2):443.

Rodnitzky RL, Levin HS, Morgan DP (1978). Effects of Ingested Parathion on Neurobehavioral Functions. Clinical Toxicology 13(3):347-359.

Sakamoto T, Sawada Y, Nishide K, Sadamitsu D, Yoshioka T, Sugimoto T, Nishii S, Kishi H (1984). Delayed neurotoxicity produced by an organophosphorous compound (Sumithion). Archives of Toxicology 56:136-138.

Seno H, Hattori H, Kumazawa T, Ishii A, Watanabe K, Suzuki O (1998). Quantitation of postmortem profenofos levels. Journal of Toxicology: Clinical Toxicology 36(1-2):63-65.

US EPA (2001). Diazinon: Review of a single dose and a 28-day dosing studies with diazinon in human volunteers. https://www3.epa.gov/pesticides/chem_search/cleared_reviews/csr_PC-057801_8-Nov-01_114.pdf (accessed 19 April 2024).

US EPA (2009). Chlorpyrifos: Special non-guideline assessment for RBC cholinesterase in humans. https://archive.epa.gov/osa/hsrb/web/pdf/1d5-science-rvw-kisicki-052709.pdf (accessed 6 February 2025).

van Gemert M, Dourson M, Moretto A, Watson M (2001). Use of Human Data for the Derivation of a Reference Dose for Chlorpyrifos. Regulatory Toxicology and Pharmacology 33:110-116.

Yoshida T, Kuroki Y (2010). Epidemiological studies of anticholinesterase pesticide poisoning in Japan, in Satoh T, Gupta RC (Eds.), Anticholinesterase Pesticides: Metabolism, Neurotoxicity, and Epidemiology. John Wiley & Sons, Inc., Hoboken, New Jersey, pp 457 – 462.

Yoshida M, Shimada E, Yamanaka S, Aoyama H, Yamamura Y, Owada S (1987). A Case of Acute Poisoning with Fenitrothion (Sumithion). Human & Experimental Toxicology 6(5):403-406.
